# Rupture signs on computed tomography, treatment, and outcome of abdominal aortic aneurysms

**DOI:** 10.1007/s13244-014-0327-3

**Published:** 2014-05-01

**Authors:** Kim-Nhien Vu, Youri Kaitoukov, Florence Morin-Roy, Claude Kauffmann, Marie-France Giroux, Éric Thérasse, Gilles Soulez, An Tang

**Affiliations:** 1 Department of Radiology, Centre hospitalier de l’Université de Montréal (CHUM), Hôpital Saint-Luc, 1058 Saint-Denis, Montréal, Québec Canada H2X 3J4; 2 Department of Radiology, Radio-Oncology and Nuclear Medicine, and Institute of Biomedical Engineering, Université de Montréal, 2900 Édouard-Montpetit, Montréal, Québec Canada H3T 1J4; 3 Department of Radiology, Centre hospitalier de l’Université de Montréal (CHUM), Hôpital Notre-Dame, 1560 Sherbrooke Est, Montréal, Québec Canada H2L 4M1; 4 Centre de recherche du Centre hospitalier de l’Université de Montréal (CRCHUM), 900 Saint-Denis, Montréal, Québec Canada H2X 0A9; 5 Department of Radiology, Centre hospitalier de l’Université de Montréal (CHUM), Hôtel-Dieu de Montréal, 3840 Saint-Urbain, Montréal, Québec Canada H2W 1T8; 6 Department of Radiology, Université de Montréal and Centre de recherche du Centre hospitalier de l’Université de Montréal (CRCHUM), Hôpital Saint-Luc, 1058 Saint-Denis, Montréal, Québec Canada H2X 3J4

**Keywords:** Abdominal aortic aneurysm (AAA), Ruptured aneurysm, Computed tomography, Endovascular aneurysm repair (EVAR), Vascular surgical procedures

## Abstract

**Objectives:**

Abdominal aortic aneurysm (AAA) rupture has a high mortality rate. Although the diagnosis of a ruptured AAA is usually straightforward, detection of impending rupture signs can be more challenging. Early diagnosis of impending AAA rupture can be lifesaving. Furthermore, differentiating between impending and complete rupture has important repercussions on patient management and prognosis. The purpose of this article is to classify and illustrate the entire spectrum of AAA rupture signs and to review current treatment options for ruptured AAAs.

**Methods:**

Using medical illustrations supplemented with computed tomography (CT), this essay showcases the various signs of impending rupture and ruptured AAAs. Endovascular aneurysm repair (EVAR) and open surgical repair are also discussed as treatment options for ruptured AAAs.

**Results:**

CT imaging findings of ruptured AAAs can be categorised according to location: intramural, luminal, and extraluminal. Intramural signs generally indicate impending AAA rupture, whereas luminal and extraluminal signs imply complete rupture. EVAR has emerged as an alternative and possibly less morbid method to treat ruptured AAAs.

**Conclusions:**

AAA rupture occurs at the end of a continuum of growth and wall weakening. This review describes the CT imaging findings that may help identify impending rupture prior to complete rupture.

***Teaching Points*:**

- *AAA rupture occurs at the end of a continuum of growth and wall weakening.*
- *Intramural imaging findings indicate impending AAA rupture.*
- *Luminal and extraluminal imaging findings imply complete AAA rupture.*
- *Some imaging findings are not specific to AAA ruptures and can be seen in other pathologies.*
- *EVAR has emerged as an alternative and possibly less morbid method of treating ruptured AAAs.*

## Introduction

Abdominal aortic aneurysm (AAA) is defined as a focal dilation of the aorta of more than 50 % of its expected diameter [2]. Found in 1 % of the population over the age of 50 [3], AAAs are responsible for 1 % of all deaths amongst Caucasian adults [4] and are the tenth leading cause of death in men over the age of 55 [5]. Risk factors include age, male gender, tobacco usage, family history, hypercholesterolaemia, and hypertension [6]. Aneurysms may progress in size as a result of gradual wall weakening [7], with rupture occurring at the end of the growth spectrum.

A ruptured AAA often leads to disastrous consequences, with 50 % of patients dying before reaching the hospital and another 40 to 50 % mortality rate for those who undergo surgery [6]. However, surgical treatment can be slightly delayed in haemodynamically stable patients who present with an impending AAA rupture. These patients still require urgent treatment, but can also benefit from preoperative imaging that optimises surgical planning and reduces mortality rates to those comparable to elective procedures (less than 5 %) [6, 8]. It is therefore crucial to recognise imaging signs of impending and complete AAA ruptures as proper identification of these rupture signs will have important repercussions on treatment promptness and prognosis.

The purpose of this review is to identify signs of impending AAA rupture before rupture is completed. This article categorises imaging findings according to location: (1) intramural, (2) luminal, and (3) extraluminal (Table 1). Intramural signs generally indicate impending AAA rupture, whereas luminal and extraluminal signs imply complete rupture. Treatment options for AAA ruptures will also be discussed.

**Table 1:** Classification of signs of impending and complete aortic aneurysm rupture according to location: intramural, luminal, and extraluminal

Location	Imaging findings	Complete rupture	Impending rupture
Intramural	Increased aneurysm size	−	+
	Rapid enlargement rate	−	+
	Focal wall discontinuity	+	+
	Hyperattenuating crescent sign	−	+
	Thrombus fissuration	−	+
	Draped aorta sign	−	+
Luminal	Aortoenteric fistula	+	−
	Aortocaval fistula	+	−
Extraluminal	Periaortic stranding	−	+
	Contrast extravasation	+	−
	Retroperitoneal haematoma	+	−
	Intraperitoneal haematoma	+	−

It must be added that an aneurysm about to rupture may not reveal any imaging findings. In addition, a painful AAA, in itself, may indicate impending rupture, even in the absence of CT imaging findings, and may justify urgent repair [6].

At our institution, computed tomography (CT) angiography using a multidetector scanner is the standard of practice for evaluating AAA. Unenhanced images are first acquired through the abdomen and pelvis. Then, a bolus-tracked CT angiography is performed using 80–120 ml of intravenous contrast agent at an injection rate of 5 ml/s. Parameters include 120 kV with auto-milliamperage, 0.50–0.75 mm collimation and 1 mm slice thickness, reconstructed every 0.9 mm. Delayed venous phase scanning performed 70–90 s after initial contrast injection can be helpful for confirmation of rupture and imaging of active contrast extravasation. Thin-slice acquisition allows for subsequent multiplanar and curved planar reformation, which are useful for planning emergency endovascular aneurysm repair (EVAR). The role of EVAR in ruptured aneurysms has been associated with lower morbidity even though this has not been confirmed in a randomised trial [9, 10].

## Intramural signs

### Increased aneurysm size

Aneurysm size is the most important predictive factor for AAA rupture [11, 12]. Following Laplace’s law, wall tension increases with vessel radius. Accordingly, rupture risk increases with size, with a 3-15 % risk per year for those with a 5–6 cm aneurysm, 10–20 % for 6–7 cm aneurysms, 20–40 % for 7–8 cm aneurysms, and 30–50 % for those with a diameter greater than 8 cm [13]. Rupture risk was shown to be substantially higher with larger AAAs, as reported in a series of patients refusing or unfit for elective repair [11].

### Rapid enlargement rate

**Fig. 1:**
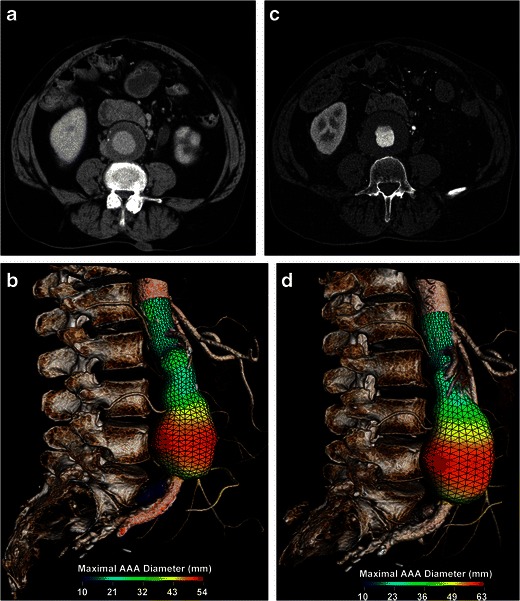
Rapid enlargement rate. **a** Axial enhanced CT and **b** 3D rendering image with a colour parametric map of a 71-year-old man shows a 5.4-cm AAA. **c** Axial enhanced CT and **d** 3D rendering image with a colour parametric map of the same patient performed a year later shows a 1 cm increase in diameter

The AAA growth rate is correlated to its diameter and to the risk of rupture [5, 14]. When smaller than 4 cm, aneurysms grow at a slower rate of 1.0–1.5 mm per year, whereas AAAs of more than 4 cm tend to have a greater growth rate of 3 mm per year [15]. With every 5 mm increase in diameter, there is a 0.5 mm per year increase in growth rate and a doubling of rupture risk [16]. Active smokers are also at higher risks of having rapidly enlarging AAAs [17]. When the aneurysm growth rate exceeds 1 cm per year, the increased rupture risk justifies elective AAA repair [6, 14] (Fig. 1).

### Focal wall discontinuity

**Fig. 2:**
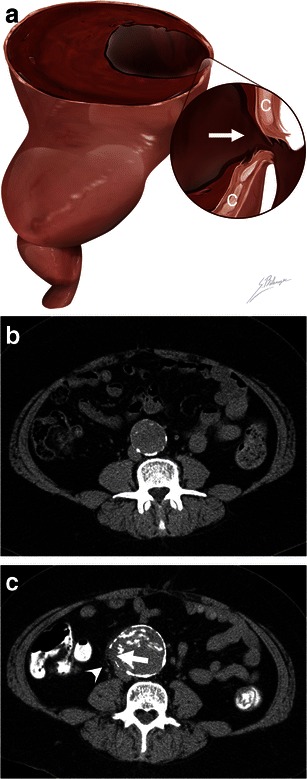
Focal wall discontinuity. **a** Illustration depicts a focal discontinuity (*white arrow*) of the calcified intima walls (*C*), indicating the AAA rupture site. **b** Axial unenhanced CT image of an asymptomatic 56-year-old man shows a 4.2 cm AAA with intact circumferential calcified walls. **c** Axial unenhanced CT image of the same patient who presented with severe lumbar pain 4 years later. The AAA now measures 5.7 cm, and a new 1 cm focal gap (*white arrow*) of the circumferential calcifications can be seen with periaortic fat infiltration near the rupture site (*white arrowhead*)

Many aneurysms are lined with circumferential wall calcifications. In impending or complete AAA rupture, a focal discontinuity of the intimal calcifications can be seen indicating the rupture site, which is most commonly observed on the posterolateral wall [18]. Found in 8 % of ruptured aneurysms [5, 19], this sign is most useful when prior intact circumferential calcified walls were documented on previous scans (Fig. 2). Alternatively, an impending rupture site can also be seen as a focal bulging of the aneurysm wall, also described as an aortic “bleb.” This corresponds histologically to inflammatory changes and a focal thinning of elastic fibres [20].

### Hyperattenuating crescent sign

**Fig. 3:**
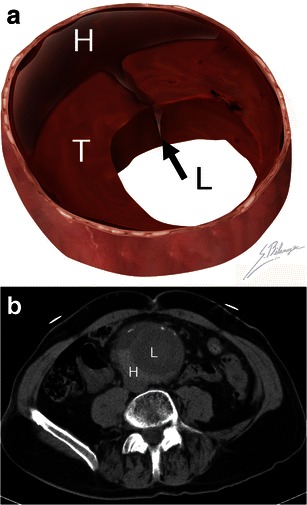
Hyperattenuating crescent sign. **a** Illustration demonstrates blood (*black arrow*) dissecting into a mural thrombus (*T*) from the aortic lumen (*L*). The resulting intramural haematoma (*H*) is crescent shaped. **b** Axial unenhanced CT of a 63-year-old man presenting with abdominal pain and a pulsating mass. A crescent (*H*) of higher attenuation than the aortic lumen (*L*) can be seen

Although intramural thrombus can be regarded as a protective factor against rupture, in fact, intrinsic metabolic activity within the thrombus weakens the underlying aortic wall [21]. When intraluminal blood dissects into the thrombus and comes into contact with the weakened aortic wall, rupture risk is increased [19, 21, 22]. The dissecting blood can be seen as a high-attenuating crescent underlying the intramural thrombus or lying within the aortic wall [7]. The attenuation of this crescent is greater than the aortic lumen on unenhanced scans, and it remains greater than the psoas muscle on enhanced scans [7]. The sensitivity and specificity of the hyperattenuating crescent sign to detect AAA rupture are 77 % and 93 %, respectively [23, 24] (Fig. 3).

### Thrombus fissuration

**Fig. 4:**
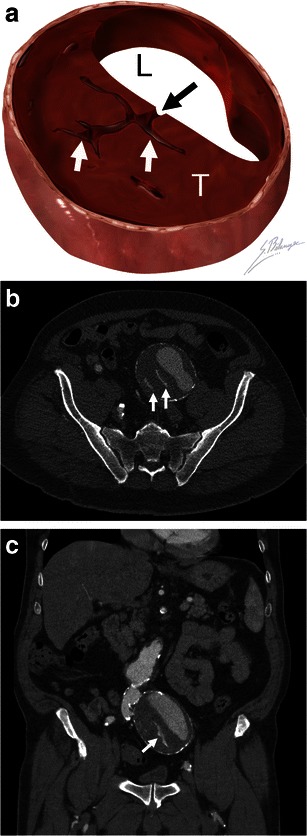
Thrombus fissuration. **a** Illustration demonstrates blood (*black arrow*) dissecting from the aortic lumen (*L*) into a mural thrombus (*T*). The infiltrating blood is seen as linear fissurations (*white arrows*). **b** Axial and **c** coronal enhanced CT of a 64-year-old man shows linear infiltrations of contrast material (*white arrows*) within the hypodense mural thrombus. The patient underwent successful emergent AAA repair before complete rupture occurred

Following the same concept described for the hyperattenuating crescent sign, blood dissection into intramural thrombus can be seen as thrombus fissuration [7]. This sign is observed on enhanced CT as linear contrast infiltrations from the patent aortic lumen through the intramural thrombus (Fig. 4). A study using mathematical models has suggested that when thrombus fissurations extend from the aortic lumen to the aneurysm wall, AAA wall tension may increase, indicating impending aneurysm rupture [25].

### Draped aorta sign

**Fig. 5:**
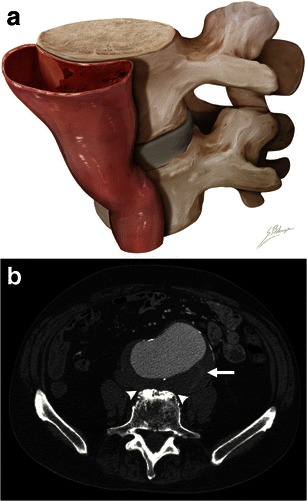
Draped aorta sign. **a** Illustration depicts loss of normal aneurysm wall convexity. The posterior wall of the aorta moulds to the anterior surface of the vertebral body. **b** Axial enhanced CT of an 85-year-old man with abdominal pain. The posterior aortic wall follows the contour of the anterior portion of the vertebra, with loss of fat planes between the aneurysm and vertebra (*white arrowheads*). Discrete thrombus fissuration (*white arrow*) is also seen. These are both signs of impending AAA rupture

A draped aorta sign can be seen in contained AAA ruptures, when the rupture site is posterior and sealed by the adjacent vertebral body [8]. The posterior wall of the aorta moulds to the anterior surface of the vertebra. Normal fat planes between the aneurysm and vertebra are lost. When the contained rupture is chronic, smooth vertebral erosions can be observed in up to 30 % of cases [8] (Fig. 5).

## Luminal signs

### Aortoenteric fistula

**Fig. 6:**
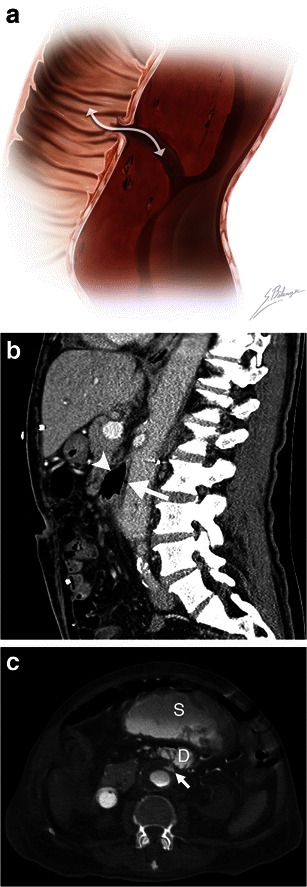
Aortoenteric fistula. **a** Illustration depicts a fistulous tract connecting a bowel loop to an aortic aneurysm. The *white double-headed arrow* shows communication between the structures allowing bowel gas to infiltrate into the aortic wall and blood to leak into the bowel lumen. **b** Sagittal enhanced CT image of a 73-year-old man demonstrates an aortoenteric fistula. Intraluminal gas (*white arrow*) is observed within the AAA, and normal fat planes between the aneurysm and the third portion of the duodenum are lost (*white arrowhead*). **c** Axial enhanced CT of an 82-year-old man who presented with massive lower gastrointestinal bleeding and a history of previously repaired AAA. Active contrast extravasation (*white arrow*) into the third portion of the duodenum (*D*) and the stomach (*S*) can be seen in this patient with an aortoenteric fistula

Aortoenteric fistulas can be due to atherosclerotic disease, without prior aortic surgery, or, much more commonly, associated with a surgically repaired aneurysm. The latter fistulas may develop long after the initial surgery [26]. Patients may present with the classic triad of known AAA, abdominal pain, and gastrointestinal bleeding. Although endoscopic examinations are often negative, CT scans may show characteristic findings. Imaging findings include intra- and extraluminal gas within the aneurysmal sac and loss of fat planes between the aneurysm and involved bowel segment. Contrast extravasation into the bowel lumen can sometimes be seen on enhanced CT when the patient is actively bleeding. The third and fourth portions of the duodenum are involved in over 50 % of aortoenteric fistulas [6]. Aortoenteric fistulas have an up to 30–40 % mortality rate [6] (Fig. 6).

### Aortocaval fistula

**Fig. 7:**
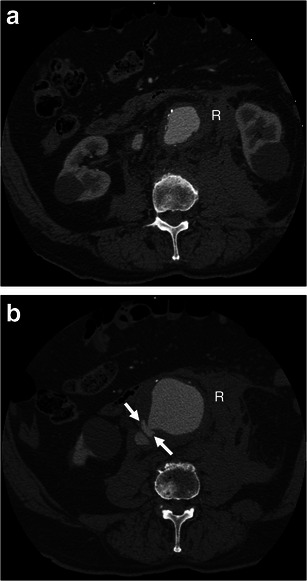
Aortocaval fistula. **a** Axial arterial phase enhanced CT shows simultaneous enhancement of the AAA and IVC in an 83-year-old man with AAA rupture and retroperitoneal haematoma (*R*). **b** Axial enhanced CT of the same patient at a lower level demonstrates active contrast extravasation (*white arrows*) from the aortic aneurysm to the IVC with loss of normal fat planes between the structures. Retroperitoneal haematoma (*R*) can also be seen

An aortocaval fistula is a rare entity that affects less than 1 % of AAAs and 2–4 % of ruptured aneurysms [27, 28]. They are associated with a 30 % mortality rate [6]. Symptoms of congestive heart failure and venous congestion can be observed clinically. On unenhanced CT, normal fat planes between the AAA and inferior vena cava (IVC) are obliterated, and the IVC may appear dilated. On arterial phase CT angiography, the AAA and IVC enhance simultaneously, whereas the renal cortex and femoral arteries show later enhancement [28] (Fig. 7).

## Extraluminal signs

### Periaortic stranding

**Fig. 8:**
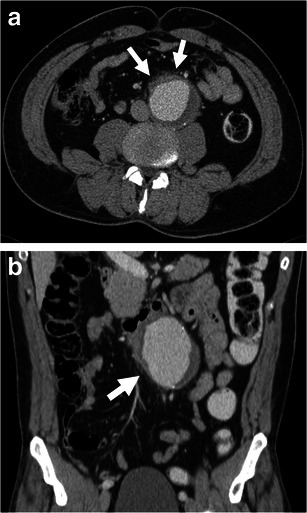
Periaortic stranding. **a** Axial and **b** coronal enhanced CT of a 51-year-old man with abdominal pain shows stranding of periaortic fat (*white arrows*) before any retroperitoneal haematoma can be seen

Periaortic fat stranding is frequently observed in impending rupture and may be the earliest sign before complete AAA rupture [5]. It represents oedema of the periaortic fat [29] and can be seen before visualisation of a retroperitoneal haematoma. Fat stranding is seen as an area of high-attenuating fat surrounding a large aneurysm [29]. It can range from a discrete and indistinct peripheral infiltration to a coarse linear pattern [29] (Fig. 8).

### Contrast extravasation

**Fig. 9:**
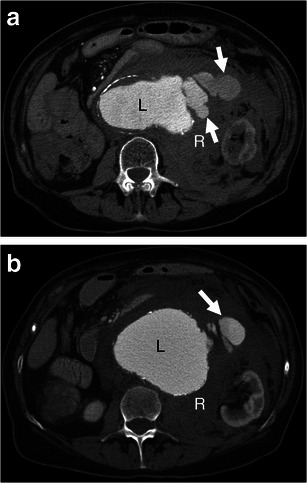
Contrast extravasation. **a**-**b** Axial enhanced CT of a 75-year-old woman demonstrates active contrast extravasation (*white arrows*) from the aneurysm lumen (*L*) into the retroperitoneal space with massive retroperitoneal haematoma (*R*)

Contrast extravasation is the most specific sign of a complete AAA rupture. On arterial phase enhanced CT, contrast material from the AAA lumen is seen escaping through the boundaries of the aneurysm wall into the retroperitoneal space. Venous phase imaging can demonstrate an expanding pool of contrast material that is increased in size compared to the arterial phase images. This sign indicates unequivocal AAA rupture (Fig. 9).

### Retroperitoneal haematoma

**Fig. 10:**
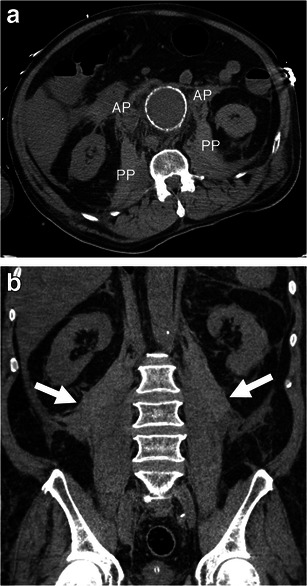
Retroperitoneal haematoma. **a** Axial unenhanced CT image of a 72-year-old man demonstrates haemorrhage involving bilateral anterior (*AP*) and posterior (*PP*) pararenal spaces as well as the area along the psoas muscles. The left kidney is displaced laterally. Attenuation of 45 HU (more than 30 HU) indicates acute haematoma. **b** Coronal unenhanced CT image of the same patient shows bilateral retroperitoneal haematomas (*white arrows)*

Given that AAAs are retroperitoneal and usually rupture their posterior and posterolateral walls [23], the most common sign of a ruptured AAA is the concomitant presence of an AAA and an adjacent haematoma in the retroperitoneal space [2, 19]. Acute haemorrhage can be seen as a high-attenuating fluid collection of 30 HU or more [5], although lower attenuation values do not exclude haematoma, especially in patients with anaemia. Bleeding may extend into multiple retroperitoneal compartments, such as the perirenal, anterior and posterior pararenal spaces, and along the psoas muscle [30]. Large haematomas may displace the kidneys laterally and the small bowel anteriorly against the anterior abdominal wall (Fig. 10).

### Intraperitoneal haematoma

**Fig. 11:**
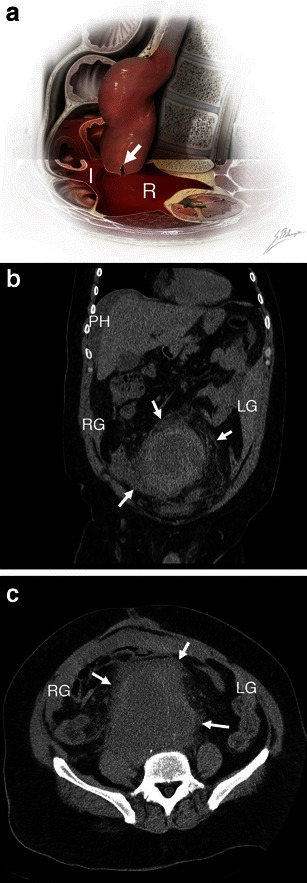
Intraperitoneal haematoma. **a** Illustration depicts AAA rupture of the left anterolateral wall (*white arrow*). Massive haemorrhage extends into both the retroperitoneal (*R*, shown as translucent red blood) and intraperitoneal (*I*, shown as more opaque blood) spaces. **b** Coronal unenhanced CT of a 51-year-old man demonstrates intraperitoneal haematoma involving the perihepatic space (*PH*), right (*RG*), and left (*LG*) paracolic gutters. Retroperitoneal haematoma (*white arrows*) is also seen. Attenuation of 60 HU indicates acute haemorrhage. **c** Axial unenhanced CT of the same patient shows right (*RG*) and left (*LG*) paracolic gutters, as well as retroperitoneal haematoma (*white arrows*)

Bleeding from a ruptured AAA may extend into the intraperitoneal compartment. Haemoperitoneum can be seen in the perihepatic space, within mesenteric folds, along paracolic gutters, or within the pouch of Douglas. Such intraperitoneal haematomas are more commonly associated with rupture of the anterior or anterolateral aortic aneurysm wall [23] (Fig. 11).

## Differential diagnosis

Some of the previously mentioned signs are not entirely specific to impending or complete AAA rupture and can be seen in other pathologies.

Intra- and extraluminal gas seen with aortoenteric fistulas can also be found in infected (mycotic) aneurysms. Such aneurysms often have a more saccular shape and irregular walls that are devoid of calcifications [5]. Occasionally, signs of primary infection sites such as vertebral osteomyelitis, pyelonephritis, or retroperitoneal abscesses may provide clues to the proper diagnosis. Clinically, these patients do not present with gastrointestinal bleeding.

Periaortic stranding can also be seen in inflammatory aneurysms, associated with autoimmune diseases (lupus, rheumatoid arthritis, giant cell arteritis) and retroperitoneal fibrosis [31]. In such cases, the aortic wall is thickened from long standing inflammation. This thickening is outside the intimal calcifications, may enhance with intravenous contrast injection, and frequently has more defined margins than periaortic haematomas. Periaortic fibrosis can extend to adjacent organs, such as the small bowel and ureters [5]. Malignancy, drug toxicity, retroperitroneal injury, and infection are also associated with retroperitoneal fibrosis and need to be included in the differential diagnosis of periaortic fibrosis [32].

Retroperitoneal haematoma can be the result of haemorrhage secondary to anticoagulation. Such haematomas are often restricted to the posterior pararenal compartment, typically intramuscular within the psoas muscle, or at a distance from the abdominal aorta. A “haematocrit effect” may also be seen as a blood-fluid level [33]. Rarely, haematomas secondary to other vascular injuries in the retroperitoneum, such as rupture of smaller lumbar, renal capsular, or inferior pancreaticoduodenal arteries or pseudoaneurysms, can mimic AAA rupture. Analysing the images for other such vascular anomalies, relating the arterial vascular anomalies to the anatomical site of the haematoma, and correlating these to the clinical and previous surgical history can help distinguish AAA rupture from other arterial branch ruptures.

## Management

AAA rupture represents a major surgical emergency that requires prompt intervention. However, an estimated 87.5 % of patients admitted to the hospital with a ruptured AAA survived longer than 2 h after their admission [34]. Furthermore, the median time interval between admission and death from AAA rupture was 10.5 h [34]. This suggests that most patients with ruptured AAAs who reach the hospital alive are haemodynamically stable enough to undergo preoperative imaging. CT angiography permits confirmation of AAA rupture and evaluation of AAA anatomy while intravenous fluid resuscitation is initiated [34]. Pre-interventional imaging also allows assessing the eligibility for an endovascular repair.

It has been reported that aneurysm repair can be slightly delayed in haemodynamically stable patients who present with symptomatic AAA without signs of complete rupture [6, 35, 36]. Urgent treatment is still warranted in these patients, who should receive proper preoperative management and aneurysm repair within 4 to 24 h of admission [6, 36]. Such management optimises preoperative conditions and reduces mortality rates to those comparable to elective procedures (less than 5 %) [6, 35, 36]. Nevertheless, in haemodynamically unstable patients with acute and complete AAA rupture, emergent and immediate treatment is mandatory.

**Fig. 12:**
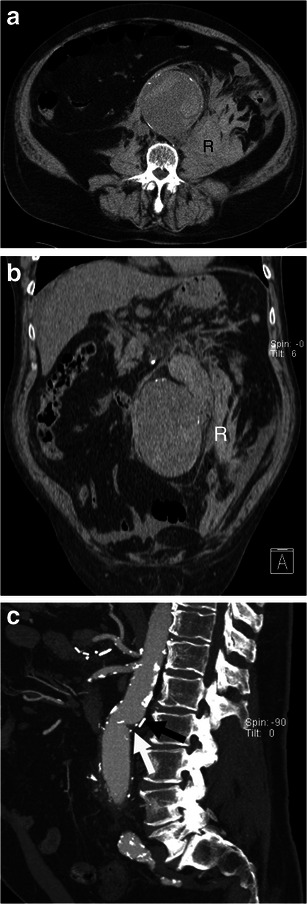
Open surgical repair. **a** Axial and **b** coronal unenhanced CT image of a 73-year-old man demonstrates ruptured AAA with a retroperitoneal haematoma (*R*). **c** Sagittal enhanced CT image of the same patient after successful open surgical repair shows the junction between the native aorta and aortic graft (*white arrow)* and a surgical clip (*black arrow*)

Open surgical repair is considered the standard treatment for AAA. Surgical repair involves an extensive abdominal incision, clamping of the proximal abdominal aorta, and suturing of a graft that excludes the aneurysm [6] (Fig. 12). Despite important advances in technology and critical care, very little or no significant reduction in mortality rates in patients with open surgical repair of ruptured AAA has been noted in over 2 decades, remaining higher than 40 % [37–40].

**Fig. 13:**
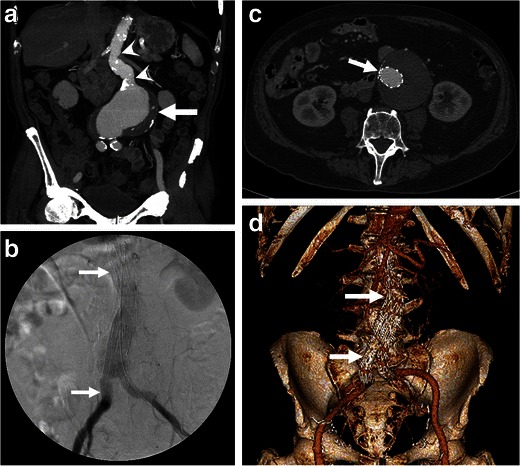
Endovascular aneurysm repair. **a** Coronal enhanced CT image of an 85-year-old man presenting with an impending AAA rupture (same as Fig. 5) demonstrates a large AAA (*white arrow*) with a long proximal neck (*white arrowheads*). **b** Fluoroscopy image, **c** axial, and **d** 3D rendering of the same patient show a deployed aorto-biiliac stent-graft (*white arrows)*

EVAR was first introduced as an alternative to open surgical AAA repair in 1986 [41]. Under fluoroscopic guidance using a transfemoral approach, a covered stent-graft bridges the proximal neck of the AAA with one (mono-iliac stent-graft) or both iliac arteries (bifurcated stent-graft) to isolate its walls from the arterial pressure (Fig. 13) [42]. This excludes the aneurysm from normal blood circulation, which leads to a thrombosis of the aneurysmal sac. If a mono-iliac stent-graft is used, a femoro-femoral bypass graft preserves opposite limb perfusion [42].

In 1994, Yusuf et al. demonstrated that EVAR could also be used in emergency situations, such as impending and even complete AAA ruptures [9, 43]. However, the logistical challenge of offering an endovascular service at any time limits the widespread use of EVAR [44]. In addition, not all patients presenting with a rupturing AAA are suitable for EVAR. Since stent-graft sizing and selection need to be adapted to patient anatomy, a relatively wide stent-graft inventory is required, which can become cumbersome logistically and financially. Pre-interventional imaging is required to evaluate aneurysm anatomical suitability for an endovascular procedure. Some favourable anatomical criteria include sufficient proximal neck length, normal proximal and distal neck diameters, limited neck angulation, and a limited amount of mural thrombus and calcification in the neck (Table 2) [6, 45–49]. This imaging also needs to assess common femoral and iliac artery anatomy as limited transfemoral access, seen in patients with small iliac arteries or severe occlusive disease, may preclude the possibility of an EVAR.

**Table 2:** Anatomic features favourable for endovascular aneurysm repair [6, 45–49]

Anatomic inclusion criteria
Proximal neck length more than 10 to 15 mm
Proximal neck diameter less than 32 mm
Iliac neck diameters less than 5 mm
Neck angulation less than 60 to 90°
Mural thrombus and calcification occupying less than 50 % of the aortic circumference

Refinement in technological equipment has allowed more flexible inclusion criteria. For example, with the use of intra-aortic balloon occlusion of the supra-renal aorta, EVAR has become available to even haemodynamically unstable patients [49]. The arrival of fenestrated stent-grafts allowed deployment of the stent-graft with coverage of the renal and superior mesenteric arteries even in patients with short proximal AAA necks, without inducing ischaemia in these aortic branch territories, and thereby expanding anatomical suitability for EVAR [50].

Unfortunately, because of differences in interventional experience levels amongst physicians, anatomical inclusion criteria for EVAR still vary widely amongst medical centres, with eligibility rates ranging from 34 to 100 % [51]. Most studies, however, report eligibility rates over 50 % [52–55].

## Outcome

A ruptured AAA often leads to disastrous consequences, with an up to 90 % mortality rate [56, 57]. Over half of the patients do not reach the hospital alive [37, 58]. Even for those who are able to undergo open surgical repair, the mortality rate remains higher than 40 % [51–53].

The arrival of EVAR for ruptured AAA has been associated with lower mortality and morbidity, reduction in blood loss, transfusion requirements, and hospital stay [59–61]. Short-term mortality rates vary from 11 to 45 % in the literature, with most studies reporting a perioperative mortality rate around 20 % [62–64]. Even in patients with haemodynamic instability, the mortality rate with EVAR has been seen to be as low as 18 % [49, 58, 63]. A study led by Bosch et al. found a reduction in the 6-month mortality rate of EVAR over open surgery by 26.5 % [48].

Such lower mortality and morbidity may be due to the use of local, as opposed to general, anaesthesia, the lower amount of blood loss, the minimally invasive nature of EVAR, and fewer extra-aortic injuries associated with open surgery [6, 49, 51].

Despite improvements in perioperative mortality rates, no studies have shown a long-term reduction in survival rates with EVAR over open surgery [65–67]. Also, an irrefutable reduction in mortality rates has not been confirmed in randomised control studies [9, 10, 44].

Finally, EVAR is associated with endoleaks, which are the most frequent long-term complications and may end up causing aneurysm rupture. CT imaging at 1 and 12 months after the initial intervention, followed by annual controls, is therefore recommended for early detection and prevention of complications, such as endoleaks [6]. If an endoleak is observed on follow-up imaging, it needs to be carefully analysed to determine its origin. Types I (sealing zone endoleak) and III (graft failure endoleak) need to be immediately addressed as they should be considered the equivalent of an untreated AAA [42]. Type II endoleaks (retrograde flow from a covered aortic branch) are the most commonly encountered endoleaks [42]. If a type II endoleak is detected, a 6-month follow-up CT is recommended [6], and re-intervention should be considered if there is progression of the aneurysm diameter. Finally, if a type V endoleak is suspected (endotension; progression of the AAA diameter with no detectable endoleak on a dual-phase CT), it should be closely followed with imaging and possibly referred for surgical conversion.

## Conclusion

AAA rupture occurs at the end of a continuum of growth and wall weakening. Although ruptured AAAs are easily diagnosed, identifying impending rupture signs can be more challenging. This review describes the CT imaging findings that may help identify impending rupture prior to complete rupture, which has important consequences on treatment and prognosis. Knowledge of alternative causes of retroperitoneal stranding and haemorrhage may also facilitate the diagnosis. EVAR has emerged as an alternative and possibly less morbid method of treating ruptured AAAs over conventional open surgical repair.
